# Synthesis of a novel mitochondrial fluorescent probe - killing cancer cells *in vitro* and *in vivo*

**DOI:** 10.3389/fphar.2025.1543559

**Published:** 2025-04-16

**Authors:** Xiaowen Yang, Yiting Zhan, Yifei Li, Xinzhuang Shen, Yuqiu Ma, Zongjun Liu, Yipeng Liu, Chengjin Liang, Xiaoyuan Zhang, Yehao Yan, Wenzhi Shen

**Affiliations:** ^1^ Shandong Provincial Precision Medicine Laboratory for Chronic Non-communicable Diseases, Institute of Precision Medicine, Jining Medical University, Jining, China; ^2^ College of Clinical Medicine, Jining Medical University, Jining, China; ^3^ Henan Key Laboratory of Immunology and Targeted Drugs, School of Laboratory Medicine, Xinxiang Medical University, Xinxiang, China; ^4^ School of Public Health, Jining Medical University, Jining, China

**Keywords:** mitochondrial fluorescent probe (MPM-1), CRC, LUNG, proliferation, apoptosis, migration

## Abstract

**Purpose:**

The global incidence and mortality rates associated with cancer are increasing annually, presenting significant challenges in oncology, particularly regarding the efficacy and toxicity of antineoplastic agents. Additionally, mitochondria are recognized for their multifaceted roles in the progression of malignant tumors. Mitochondrial-targeting drugs offer promising avenues for cancer therapy. This study focuses on the synthesis of a mitochondrial fluorescent probe, designated Mitochondrial Probe Molecule-1 (MPM-1), and evaluates its anti-tumor effects on colon cancer (CRC) and lung cancer (LUNG) both *in vitro* and *in vivo*.

**Methods:**

Mito Tracker Green FM staining was performed to investigate the subcellular location of MPM-1. Cell cycle assay, colony formation, EdU, assay of cell apoptosis, wound healing assay, and trans-well migration assay were utilized to confirm anticancer properties of MPM-1 *in vitro*. Using a xenograft mouse model, the effects of MPM-1 in tumor treatment were also identified. RNA-seq and Western blot were performed to examine the underlying mechanism of MPM-1.

**Results:**

The findings indicate that MPM-1 selectively targets mitochondria and exerts inhibitory effects on CRC and LUNG cells. Specifically, MPM-1 significantly reduced the proliferation and migration of lung cancer cell lines A549 and H1299, as well as colon cancer cell lines SW480 and LOVO, with IC50 values of 4.900, 7.376, 8.677, and 7.720 µM, respectively, while also promoting apoptosis. RNA-seq analysis revealed that MPM-1 exerts its broad-spectrum anticancer effects through interactions with multiple signaling pathways, including mTOR, Wnt, Hippo, PI3K/Akt, and MAPK pathways. Additionally, *in vivo* studies demonstrated that MPM-1 effectively inhibited tumor progression.

**Conclusion:**

In summary, MPM-1 demonstrates the ability to inhibit the growth of CRC and LUNG by targeting mitochondria and modulating several signaling pathways that attenuate tumor cell migration and proliferation while promoting apoptosis. This research underscores the potential of MPM-1 as a tumor suppressor and lays a robust foundation for the future development of innovative anticancer therapies that target mitochondrial functions.

## 1 Introduction

Lung cancer (LUNG) (12.7%), breast cancer (10.9%), colorectal cancer (CRC) (9.7%), and stomach cancer (7.81%) are the most prevalent cancer types overall (Hazafa et al., 2020). Both colorectal and lung cancers are characterized by elevated mortality rates and poor survival outcomes (Schabath and Cote, 2019), are strongly associated with genetic and environmental risk factors, and have a high propensity to spread (Dornblaser et al., 2024; Hanna et al., 2006). Consequently, there is an urgent need for improved screening and treatment modalities. Current therapeutic approaches typically encompass molecular targeted therapy, chemotherapy, radiation, and surgical intervention (Alexander et al., 2020). However, the prognosis remains unfavorable, as evidenced by low 5-year survival rates (Li et al., 2023). Furthermore, chemotherapy is associated with a high recurrence rate and considerable toxicity (Gupta et al., 2019). Therefore, there is an imperative to develop innovative anticancer agents to meet the pressing therapeutic needs of patients with tumors.

In recent years, numerous bifunctional mitochondrial fluorescent probes derived from organic small molecules have been developed. These probes are designed to specifically target mitochondria and facilitate the detection of various active small molecules within these organelles. They have proven effective in the detection and visualization of multiple active species present in mitochondria, including reactive oxygen species, reductive species, metal ions, protons, and anions, among others. Given the significant association between mitochondrial function and the onset and progression of cancer, these probes have emerged as critical tools for cancer prevention and treatment (Neuzil et al., 2007). For instance, there are mitochondria-targeted fluorescent probes that operate based on redox reactions, as well as “switched” fluorescent probe nanoparticle (PFN) delivery systems that utilize lactic acid-glycolic acid copolymers (PLGAs) to assess tumor regions through switched fluorescence, thereby enabling imaging of tumor cells and tissues. Additionally, a study introduced a series of novel near-infrared (NIR) probes featuring a donor-acceptor (D-A) structure, employing thiopyran salt heterocycles as electron acceptors (Zhou et al., 2020). The findings indicated that all three probes—3k-PEG, H4-PEG, and H4-PEG-PT—exhibited mitochondrial targeting capabilities, highlighting the substantial potential of thiopyran compounds for mitochondrial NIR imaging. Consequently, mitochondrial fluorescent probes play a vital role in both the diagnosis and treatment of tumors.

The activity of signaling pathways is significantly altered in cancer cells, influencing a range of biological processes such as growth, proliferation, invasion, apoptosis, and metastasis (Vaghari-Tabari et al., 2021). The hyperactivation of the mTOR pathway, resulting from the activation of PI3K/Akt, enhances glucose uptake and promotes glycolysis, both of which are directly associated with carcinogenesis (Bauer et al., 2005; Elstrom et al., 2004; Rathmell et al., 2003). The MAPK cascade serves as a vital signaling network that regulates various cellular functions, including stress responses, apoptosis, differentiation, and proliferation (Fang and Richardson, 2005). The Hippo pathway plays a significant role in organ development, tissue regeneration, wound healing, immune regulation, and epithelial homeostasis. Dysregulation of the Hippo pathway and the activity of YAP/TAZ-TEAD have been implicated in numerous diseases, including cancers (Dey et al., 2020; Sanchez-Vega et al., 2018). Additionally, the dysregulation of the Wnt pathway has a multifaceted role in nearly all stages of tumorigenesis across various cancer types (Zhou et al., 2022). Consequently, targeting these signaling pathways presents a rational approach for cancer treatment.

In the present study, we synthesized a mitochondrial fluorescent molecular probe (MPM-1) and utilized several assays, including the CCK8 cell viability assay, cell cycle analysis, EdU assay, wound healing assay, apoptosis assay, and *in vivo* xenograft models, to demonstrate that MPM-1 effectively inhibits tumor cell proliferation and migration while promoting apoptosis in both *in vivo* and *in vitro* settings. Furthermore, through RNA sequencing, we established that MPM-1 exerts a broad-spectrum anticancer effect by modulating multiple signaling pathways, including PI3K/Akt, Hippo, Wnt, mTOR, and MAPK. Our findings expand the potential applications of MPM-1 in antitumor therapy and provide a theoretical basis for the development of novel anticancer agents.

## 2 Results

### 2.1 Synthesis of compound MPM-1

**FIGURE 1:**
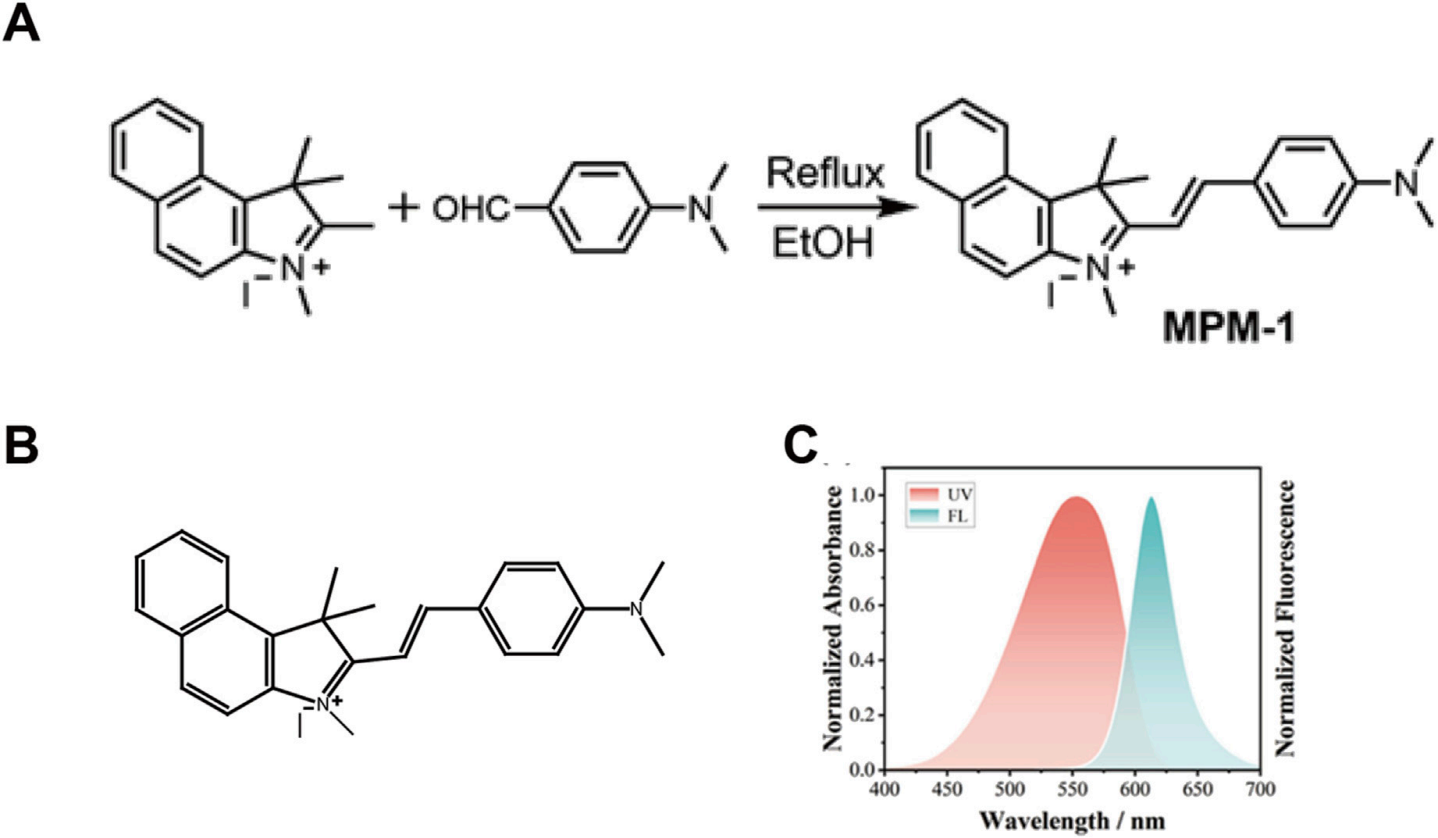
Synthesis of Compound MPM-1 **(A)** the synthetic pathway employed for the production of probe MPM-1. **(B)** The molecular structure of MPM-1. **(C)** The normalized absorbance and fluorescence characteristics of MPM-1 in dimethylformamide (DMF) solvent, with excitation at 500 nm, a slit width of 10/10, a scanning speed of 300 nm per minute, and a voltage of 700 V.

The synthesis of the mitochondria-targeting drug, mitochondrial fluorescent molecular probe (MPM-1), was conducted following methodologies established in prior study (19). A mixture of 1,1,2,3-tetramethyl-1H-benzo [e]indol-3-ium (1 mmol, 351.0 mg) and 4-(dimethylamino) benzaldehyde (1.1 mmol, 164.0 mg) was dissolved in 15 mL of ethanol (EtOH) and subjected to reflux for a duration of 8 h in the presence of piperidine (Figure 1A). The molecular structure of MPM-1 is depicted in Figure 1B. Subsequently, the product was purified using column chromatography with a solvent system of methanol/dichloromethane (MeOH/DCM) in a volume ratio of 1:15, yielding a final product with a 51% yield. The ultraviolet-visible (UV-vis) and fluorescence spectra of MPM-1 were recorded and are presented in Figure 1C. The absorption and fluorescence peaks of MPM-1 were observed at 553 nm and 613 nm, respectively, which are advantageous for bioimaging applications.

### 2.2 MPM-1 inhibits cancer cell proliferation by targeting mitochondria

**FIGURE 2:**
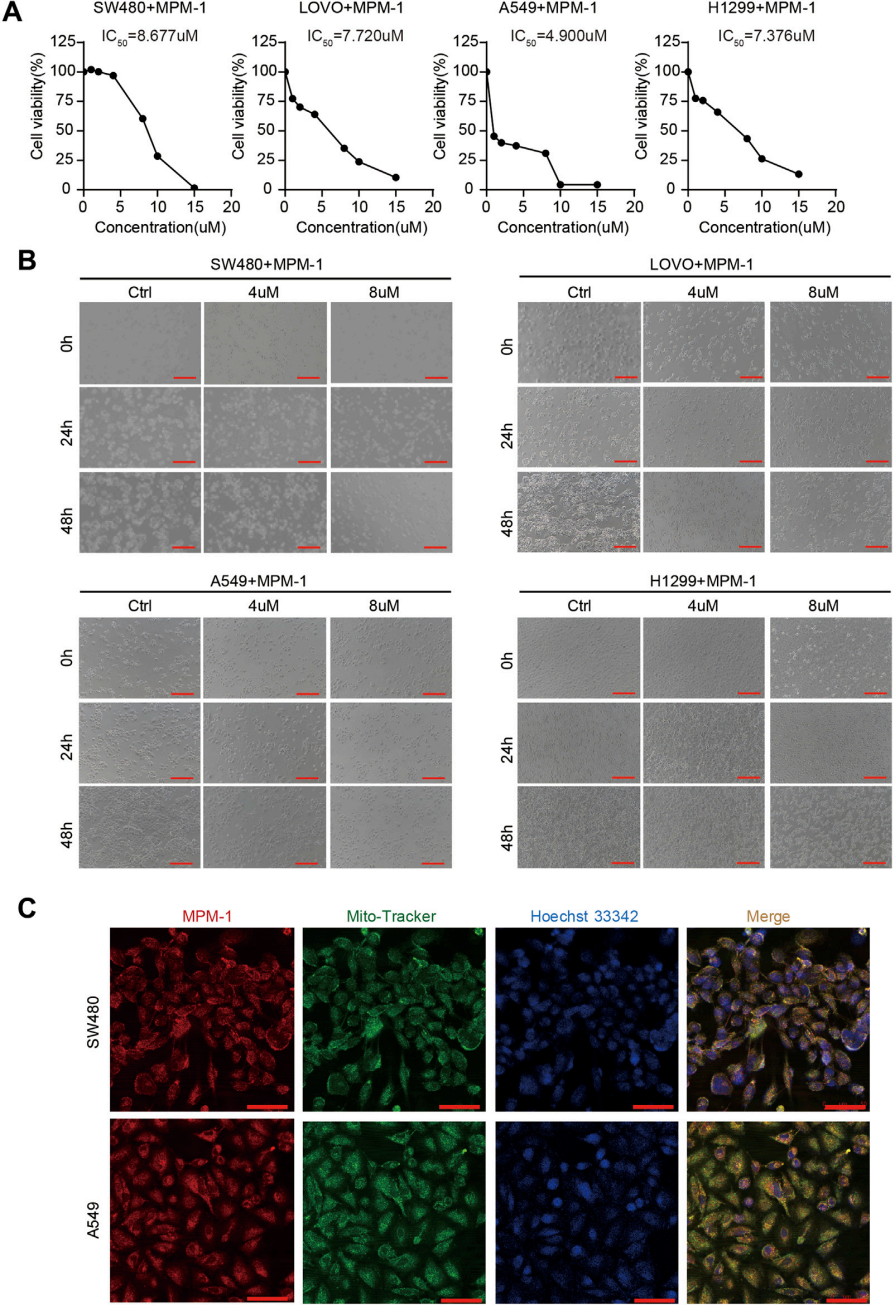
MPM-1 suppresses cancer cells by acting on mitochondria **(A)** SW480, LOVO, A549, and H1299 cell lines were treated with concentrations ranging from 0 to 15 µM of MPM-1 for a duration of 24 h, after which cell viability was assessed using a CCK-8 assay, and the half-maximal inhibitory concentration (IC50) was determined. **(B)** The morphological alterations in SW480, LOVO, A549, and H1299 cells following treatment with DMSO, as well as 4 μM and 8 µM of MPM-1 for 24 and 48 h, with observations made under a microscope (scale bar: 50 μm). **(C)** Following a 24-h treatment with MPM-1, cells were incubated with Mito-Tracker Green FM working solution at 37°C for 20 min, followed by Hoechst 33,342 working solution at 37°C for 5 min, and the localization of MPM-1 was visualized using confocal microscopy (scale bar: 20 μm).

In this study, MPM-1 was administered at concentrations ranging from 0 to 15 µM to non-small cell lung cancer cell lines A549 and H1299, as well as colon adenocarcinoma cell lines SW480 and LOVO. The calculated IC50 values for A549 and H1299 were found to be 4.900 µM and 7.376 µM, respectively, while SW480 and LOVO exhibited IC50 values of 8.677 and 7.720 µM, as illustrated in Figure 2A. Notably, A549 cells demonstrated a higher sensitivity to MPM-1 compared to the other cancer cell lines, indicating variability in the responsiveness of different cell types to this compound. Morphological assessments (Figure 2B) revealed that treatment with 4 µM MPM-1 resulted in the narrowing and elongation of A549 and H1299 cells, as well as SW480 and LOVO cells. In contrast, exposure to 8 µM MPM-1 led to a rounding and darkening of the cells, accompanied by a loss of normal morphology, membrane fragmentation, and subsequent cell death. The mechanism of action of MPM-1 was further explored using Mito-Tracker Green FM, which demonstrated that the red fluorescence of MPM-1 coincided with the green fluorescence of Mito-Tracker, indicating selective accumulation in the mitochondria (Figure 2C). These findings suggest that MPM-1 effectively penetrates the cell membrane and targets the mitochondria to exert its anti-cancer effects.

### 2.3 MPM-1 inhibits tumor cell proliferation *in vitro*

**FIGURE 3:**
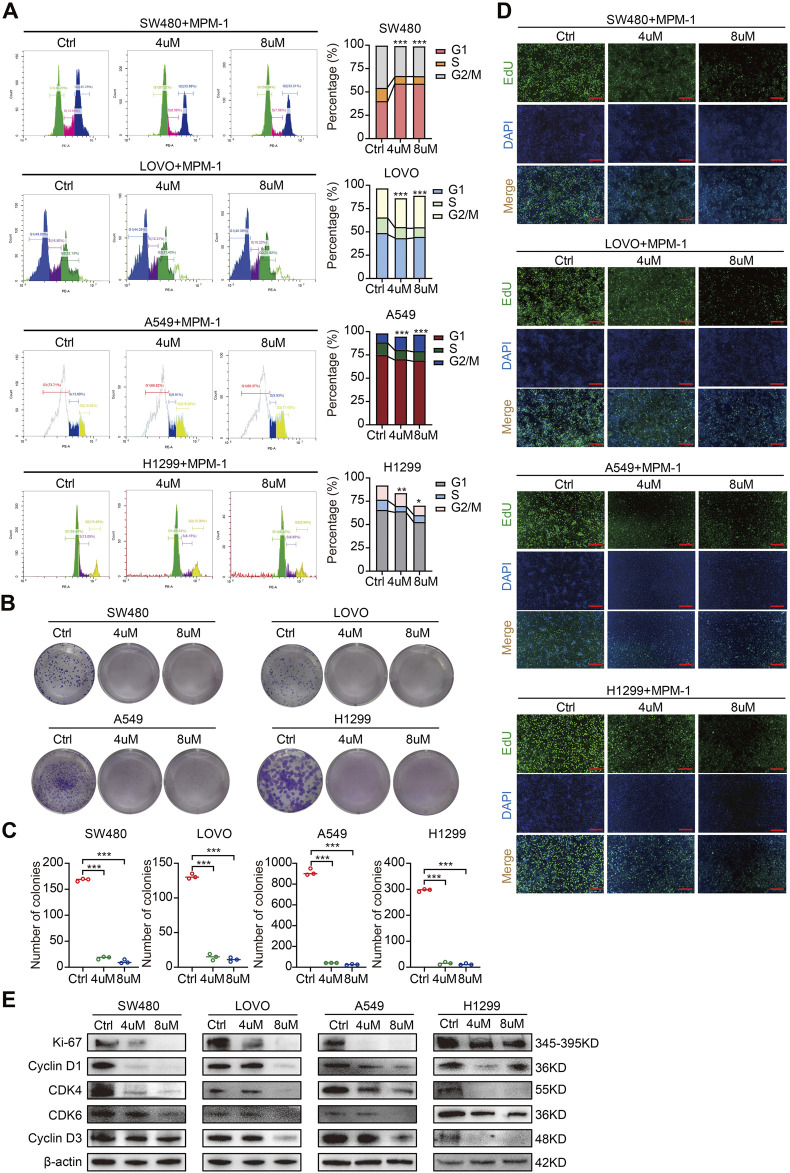
MPM-1 inhibits tumor cell proliferation *in vitro* **(A)** DMSO, 4uM and 8uM MPM-1 were administered to SW480, LOVO, A549, and H1299 cell lines for a duration of 24 h to evaluate the cell cycle dynamics. The sample size was N = 3, with statistical significance denoted as *p < 0.05, **p < 0.01, and ***p < 0.001. **(B)** SW480, LOVO, A549, and H1299 cells were incubated with DMSO, 4uM and 8uM MPM-1 for 14 days, and a colony forming assay was performed to test the effect of MPM-1 on cell proliferation. **(C)** Quantitative analysis of colony forming assay. N = 3, ***p < 0.001. **(D)** EdU assay was performed to detect the proliferative ability of SW480, LOVO, A549, and H1299 cells at different concentrations of MPM-1and DMSO. Scale bar: 50 μm. **(E)** The expression levels of Ki-67, CDK4, CDK6, Cyclin D1, and Cyclin D3 were analyzed via Western blotting, with β-actin serving as a loading control.

To elucidate the functional role of MPM-1 in tumor cell proliferation, we conducted a cell cycle assay. The results, as illustrated in (Figure 3A) indicate that MPM-1 significantly inhibits the proliferation of colon cancer cells (SW480, LOVO) and lung cancer cells (A549, H1299) by decreasing the proportion of cells in the S phase following treatment with 4 and 8 µM of MPM-1 for a duration of 24 h. To further evaluate the impact of MPM-1 on cellular proliferation capacity, we performed a colony formation assay. As shown in Figures 3B, C, MPM-1 markedly diminished the colony formation ability of SW480, LOVO, A549, and H1299 cells. Additionally, the EdU assay corroborated these findings, demonstrating that MPM-1 reduced the proliferative capacity of SW480, LOVO, A549, and H1299 cells (Figure 3D). Western blot analysis (Figure 3E) further substantiated these results, indicating that MPM-1 treatment led to a reduction in the expression of proliferation-associated proteins, including Ki-67, CDK4, CDK6, Cyclin D1, and Cyclin D3, in SW480, LOVO, A549, and H1299 cells. Collectively, these findings suggest that MPM-1 effectively suppresses tumor cell proliferation and downregulates the expression of proteins associated with cellular proliferation *in vitro*.

### 2.4 MPM-1 inhibits tumor cell migration *in vitro*

**FIGURE 4:**
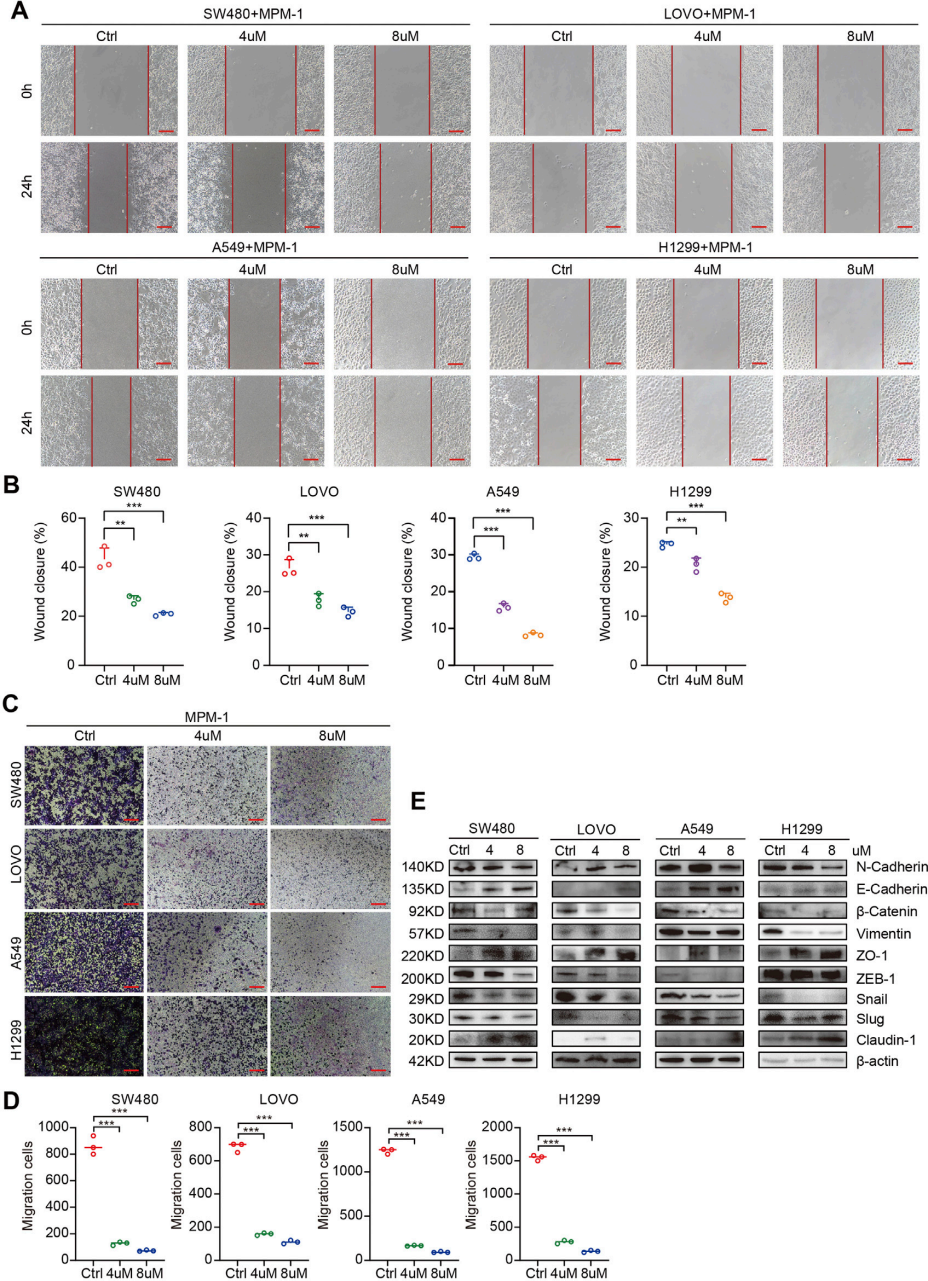
MPM-1 inhibits migration of CRC and LUNG cells **(A)** Wound healing assay was used to observe the cell migration of SW480, LOVO, A549, and H1299 cells treated with DMSO, 4uM and 8uM of MPM-1. Scale bar: 50 μm. **(B)** Quantitative analysis of cell migration rate. N = 3, **p < 0.01, ***p < 0.001. **(C)** Trans-well migration assay was performed to explore the migration ability of cells after MPM-1 treatment and DMSO. Scale bar: 50 μm. **(D)** Quantitative analysis of Trans-well migration assay. N = 3, ***p < 0.001. **(E)** The expression of EMT-related molecular markers was analyzed using Western blot, with β-actin serving as a loading control.

We also investigated the effects of MPM-1 on the migratory capacity of tumor cells by treating SW480, LOVO, A549, and H1299 cell lines with concentrations of 4 μM and 8 µM of MPM-1 for a duration of 24 h. The results from the wound healing assay indicated that MPM-1 significantly inhibited tumor cell migration in a dose-dependent manner (Figures 4A, B). To further elucidate the impact of MPM-1 on cell migration, we employed the Trans-well migration assay. Our findings revealed a marked reduction in the number of cells migrating to the lower chamber as the concentration of MPM-1 increased, suggesting a diminished migratory capacity of the tumor cells (Figures 4C, D). In alignment with these observations, Western blot analysis (Figure 4E) demonstrated that treatment with MPM-1 led to a decrease in the expression levels of N-cadherin, β-Catenin, Vimentin, Snail, Slug, and ZEB1, while concurrently increasing the expression of E-cadherin, ZO-1, and Claudin-1. In summary, our findings indicate that MPM-1 effectively inhibits the migration of colon and lung cancer cells.

### 2.5 MPM-1 induces CRC and LUNG cell apoptosis

**FIGURE 5:**
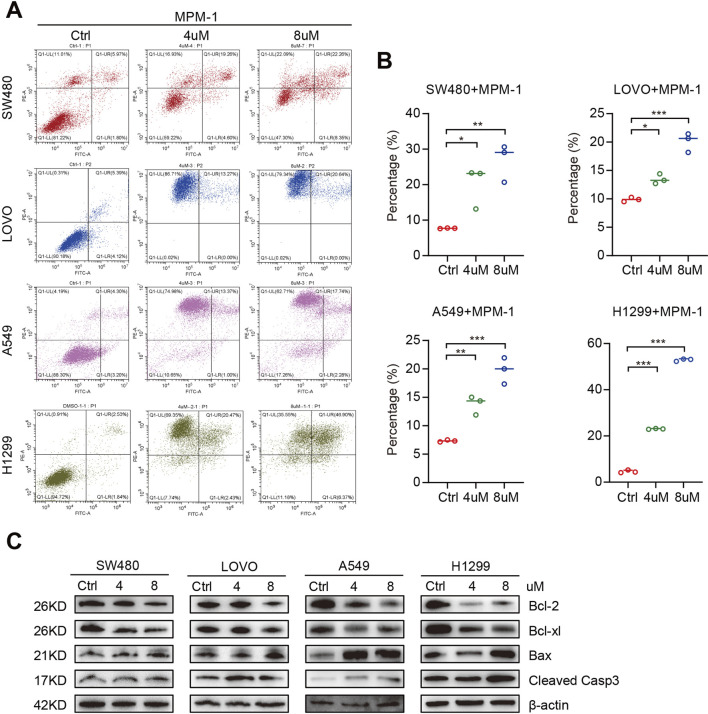
**(A)** DMSO, 4uM and 8uM MPM-1 were administered to SW480, LOVO, A549, and H1299 cell lines for a duration of 24 hours. Subsequently, propidium iodide (PI) and Annexin V staining were conducted to assess the impact of MPM-1 and DMSO on apoptotic processes. **(B) **Quantitative analysis of cell apoptosis percentage. The experiments were performed with a sample size of N = 3, with statistical significance denoted as *p <0.05, **p <0.01, and ***p <0.001. **(C)** The expression levels of Cleaved Caspase-3, Bax, Bcl-2, and Bcl-xl were evaluated in cells treated with 4 μM and 8 μM MPM-1, with β-actin utilized as a loading control.

We utilized an apoptosis assay to investigate the effects of MPM-1 on apoptotic processes. The results, as illustrated in Figures 5A, B, indicated a significant increase in the number of apoptotic cells in SW480, LOVO, A549, and H1299 cell lines when co-incubated with concentrations of 4 μM and 8 µM of MPM-1, compared to the control group. Additionally, Western blot analysis was employed to assess the expression levels of Bax, Bcl-2, Bcl-xl, and cleaved caspase 3. The data revealed that MPM-1 led to a reduction in the expression of the anti-apoptotic proteins Bcl-xl and Bcl-2, while simultaneously enhancing the expression of the pro-apoptotic proteins Bax and cleaved caspase 3 (Figure 5C). These findings suggest that MPM-1 effectively induces apoptosis in lung and colorectal cancer cells *in vitro*.

### 2.6 MPM-1 demonstrates broad-spectrum anticancer effects through multi-pathway synergy

**FIGURE 6:**
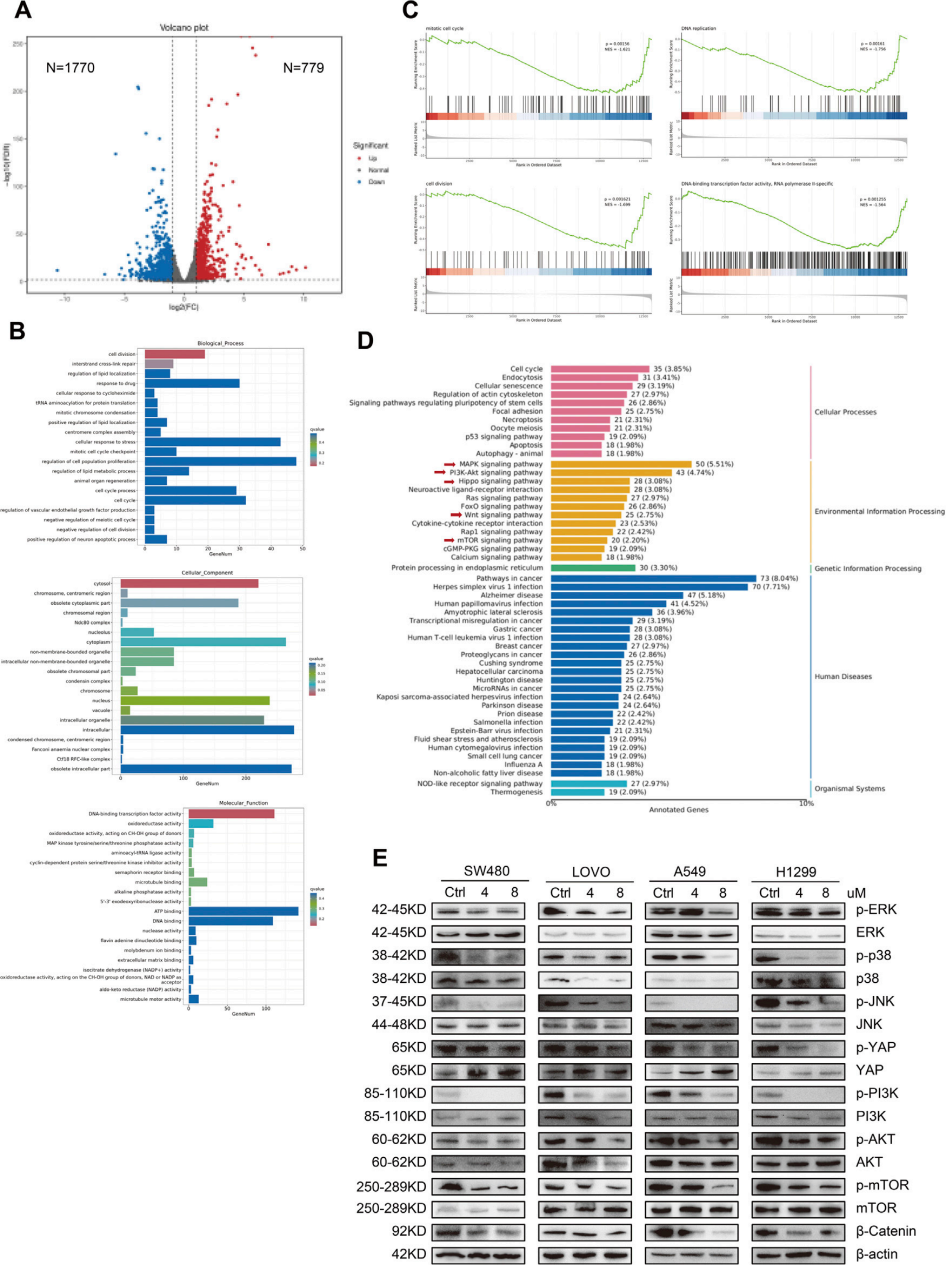
MPM-1 demonstrates extensive anticancer properties by engaging in synergistic interactions across multiple pathways **(A)** The results of the RNA sequencing analysis are presented in the form of a volcano plot. **(B)** Gene Ontology (GO) analysis revealed the impact of MPM-1 on cellular proliferation, its mechanism of action, and associated biological activities. **(C)** Gene Set Enrichment Analysis (GSEA) was conducted on the RNA sequencing data. **(D)** The Kyoto Encyclopedia of Genes and Genomes (KEGG) analysis of the RNA sequencing results is also provided. **(E)** The expression levels of various proteins, including phosphorylated and non-phosphorylated forms of ERK, P38, JNK, YAP, PI3K, AKT, mTOR, and β-Catenin, were assessed using Western blotting, with β-actin serving as a loading control.

In our investigation, we employed RNA sequencing to elucidate the molecular mechanisms by which MPM-1 inhibits migration and proliferation while promoting apoptosis in colon and lung cancer cells. The analysis of volcano plots (Figure 6A) revealed that MPM-1 treatment in SW480 cells resulted in the upregulation of 779 differentially expressed genes (DEGs) and the downregulation of 1,770 DEGs. The biological mechanisms associated with the anticancer activity of MPM-1 are illustrated in Figure 6B. Notably, MPM-1 is involved in regulating the cell cycle and the proliferation of cell populations. Its subcellular localization is predominantly intracellular, particularly within the cytoplasm and other intracellular compartments, which is essential for its anticancer effects. Furthermore, MPM-1 is associated with DNA and ATP binding, suggesting its potential role as a mitochondrial probe. Gene Set Enrichment Analysis (GSEA) consistently indicated (Figure 6C) that MPM-1 exhibits specificity for RNA polymerase II and negatively impacts processes such as the cell cycle, DNA replication, and cell division, while also exhibiting activities characteristic of DNA-binding transcription factors. KEGG pathway enrichment analysis indicated that the biological functions of MPM-1 are contingent upon the synergistic interaction of multiple signaling pathways, including the mTOR, PI3K/Akt, Hippo, Wnt, and MAPK pathways (Figure 6D). To validate the involvement of key molecules within these signaling pathways, we conducted Western blot analyses. The results (Figure 6E) indicated that while the expression levels of ERK, P38, JNK, YAP1, PI3K, AKT, and mTOR were either slightly elevated or remained stable, the phosphorylated forms of these proteins (p-ERK, p-P38, p-JNK, p-YAP1, p-PI3K, p-AKT, p-mTOR) and β-Catenin exhibited reduced expression. These findings suggest that MPM-1 utilizes a diverse array of signaling pathways, including MAPK, PI3K/Akt, Hippo, Wnt, and mTOR, to exert its broad-spectrum anticancer effects.

### 2.7 MPM-1 inhibits xenograft tumor progression *in vivo*

**FIGURE 7:**
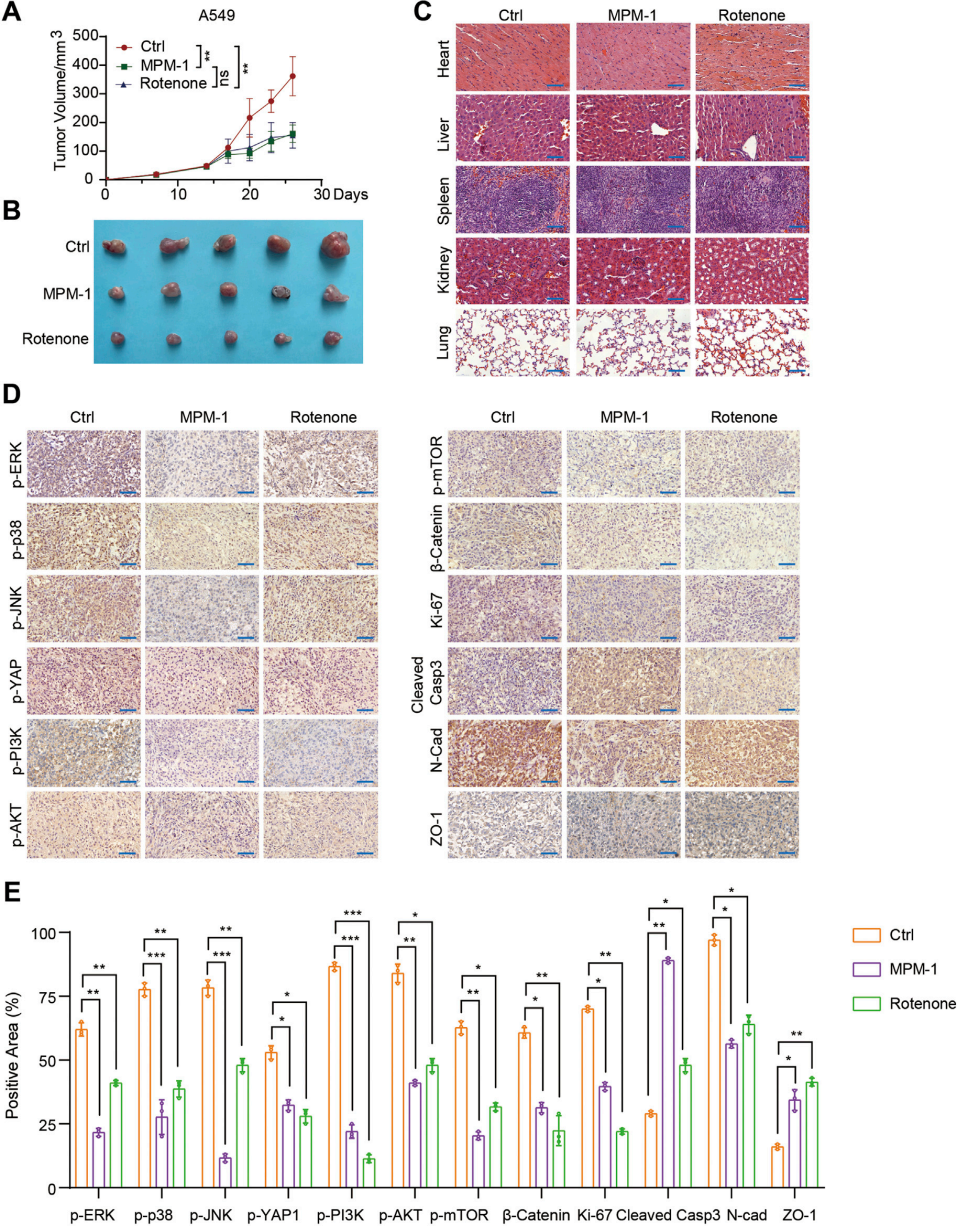
MPM-1 inhibits lung cancer xenograft tumor progression *in vivo* **(A)** A549 cells were administered via injection into the fourth fat pad of nude mice. On the 14th day post-injection, three groups of mice received intraperitoneal injections of DMSO, MPM-1 (1 mg/kg), and Retenone (1 mg/kg), respectively, and tumor growth curves were subsequently generated. The sample size was N = 5, with “ns” indicating non-significance, and statistical significance was denoted as **p < 0.01. **(B)** Tumors excised from the various groups of mice are presented. **(C)** Photomicrographs of HE staining of tissue sections from the heart, liver, spleen, lung, and kidney of the mice are displayed, with a scale bar of 50 μm. **(D)** IHC staining was performed to assess the expression levels of p-ERK, p-P38, p-JNK, p-YAP1, p-PI3K, p-AKT, p-mTOR, β-Catenin, Ki-67, N-cadherin, Cleaved Caspase-3, and ZO-1 in the tumors from the different groups of mice, with a scale bar of 50 μm. **(E)** The quantification of the various proteins was conducted through IHC detection, with N = 5, and statistical significance indicated as *p < 0.05, **p < 0.01, and ***p < 0.001.

MPM-1 has been shown to inhibit cell proliferation and migration while promoting apoptosis, thereby impeding the progression of lung and colon cancer *in vitro*. To validate these findings *in vivo*, we utilized xenograft mouse models. A549 cells were injected into the fourth fat pad of nude mice, and on the 14th day, we administered intraperitoneal injections of DMSO, MPM-1 (1 mg/kg), and Retenone (1 mg/kg) to three distinct groups of mice. The results indicated that the tumor growth rate and volume in the MPM-1 and Retenone groups were significantly lower than those observed in the control group, as depicted in Figures 7A, B. Histological examination (HE staining) revealed that MPM-1 did not cause substantial damage to the heart, liver, spleen, lung, or kidney tissues of the mice (Figure 7C). Immunohistochemical (IHC) analysis demonstrated that MPM-1 significantly reduced the positive area proportions of p-ERK, p-P38, p-JNK, p-YAP1, p-PI3K, p-AKT, p-mTOR, β-Catenin, Ki-67, and N-cadherin within the tumors. Conversely, there was an increase in the positive area proportions of Cleaved Caspase-3 and ZO-1, consistent with the *in vitro* findings (Figures 7D, E).

To further validate the aforementioned findings, colon cancer SW480 cells were administered into the fourth fat pad of nude mice. On day 15 post-injection, the mice were divided into three distinct groups and received intraperitoneal injections of either DMSO, MPM-1 at a dosage of 0.5 mg/kg, or MPM-1 at a dosage of 1 mg/kg. The results indicated that the tumor growth rate and volume in the groups treated with MPM-1 (0.5 mg/kg and 1 mg/kg) were significantly reduced compared to the control group, as illustrated in Supplemental Figures 1A, 1B. Furthermore, histological analysis via hematoxylin and eosin (HE) staining revealed that MPM-1 did not induce significant damage to cardiac, hepatic, splenic, pulmonary, or renal tissues in the mice, as depicted in Supplemental Figure 1C.

In summary, MPM-1 demonstrates a significant capacity to impede the progression of ectopic lung and colorectal cancers *in vivo*, exhibiting therapeutic efficacy that is comparable to that of Retenone in the management of lung cancer.

### 2.8 Proposed model of MPM-1 in tumor progression inhibition

**FIGURE 8:**
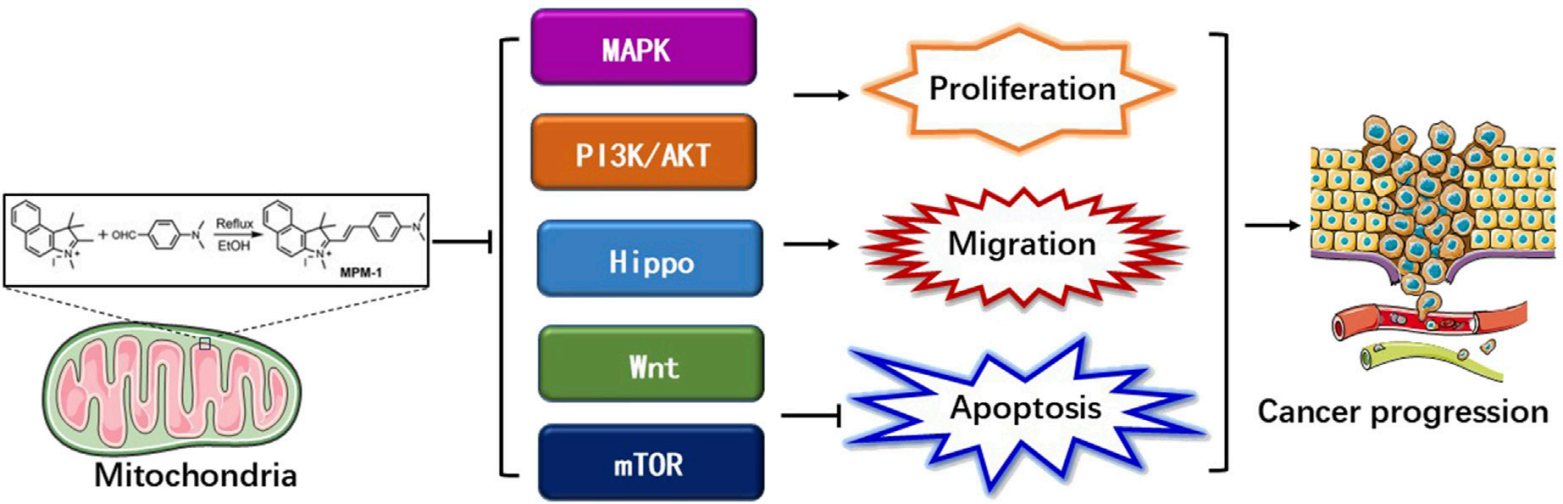
Proposed model of MPM-1 in inhibiting tumor progression.

In light of the aforementioned findings, we present the following model (Figure 8): we have developed a mitochondrial fluorescent probe, designated MPM-1, which specifically targets mitochondria. This probe has been shown to inhibit various signaling pathways, including MAPK, PI3K/AKT, Hippo, mTOR, and Wnt. Furthermore, MPM-1 effectively suppresses tumor cell proliferation and migration while promoting apoptosis both *in vitro* and *in vivo*, ultimately leading to the inhibition of tumor progression.

## 3 Discussion

The global incidence and mortality rates associated with cancer are experiencing a rapid increase annually (Wang et al., 2022). The World Health Organization (WHO) has projected that cancer will emerge as the leading cause of death worldwide in 2020, with an estimated 19.3 million new cases and approximately 10 million deaths (Bray et al., 2018). To enhance the survival rates of patients diagnosed with tumors, current clinical interventions primarily involve a combination of surgical procedures, radiotherapy, chemotherapy, immunotherapy, and targeted drug therapies. Nevertheless, the cytotoxic effects and drug resistance resulting from these treatments continue to adversely affect patient survival rates (Nguyen et al., 2021). Importantly, the early detection and treatment of tumors can significantly improve patient prognoses. Consequently, there is an urgent necessity for the development of novel pharmacological agents and diagnostic markers for individuals affected by cancer.

The exploration of cancer chemotherapeutics utilizing mitochondria-targeting molecules has garnered significant attention in recent research (Biasutto et al., 2010), given the critical and multifaceted role of mitochondria in the progression of malignant tumors (Zong et al., 2016). Furthermore, the investigation reveals that 5BMF, a novel fluorescent lipophilic cation (DLC) that accumulates in mitochondria and exhibits low toxicity alongside markedly enhanced anticancer properties, can serve as a fluorescent imaging probe for tumor visualization (Chen et al., 2019). Research indicates that the proliferation of colon cancer cells is specifically inhibited at submicromolar concentrations by nitroxides that target mitochondria (Kalyanaraman et al., 2018). Our findings demonstrate that the mitochondrial fluorescence probe MPM-1 operates synergistically with various signaling pathways to inhibit tumor cell migration and proliferation while facilitating apoptosis, both *in vivo* and *in vitro*. Consequently, MPM-1 holds promise as a potential therapeutic agent for tumors, contributing to the advancement of cancer diagnostics and treatment, and providing valuable insights for the future development of innovative fluorescence probes for clinical imaging.

Nuclear-encoded genes play a crucial role in regulating mitochondrial function through positive signaling mechanisms that enhance mitochondrial activity or stimulate mitochondrial biogenesis in response to cellular requirements. Key regulators of this process include PGC1α, NRF1, and other nuclear-encoded transcription factors (Quirós et al., 2016). This regulatory dynamic can be viewed as a reciprocal signaling process, wherein the nucleus receives feedback from the mitochondria. Such reverse signaling initiates transcriptional reprogramming that facilitates metabolic adaptability in response to various mitochondrial challenges, including intraprotein homeostatic stress, energy deficiency, and increased levels of reactive oxygen species (English et al., 2020; Smiraglia et al., 2008). The signaling interactions between the nucleus and mitochondria are predominantly governed by SIRT1, AMPK, and ATFS-1 (Quirós et al., 2016). A multitude of studies have indicated that stress signals and mitochondrial metabolites, such as α-ketoglutarate, acetyl coenzyme A, NAD^+^, and methionine, can act as signaling molecules that regulate epigenetic modifications, modify the expression of metabolism-related genes, and ultimately maintain intracellular homeostasis (Haws et al., 2020; Zhu et al., 2022). Our research indicates that MPM-1 targets mitochondria, inhibits tumor cell migration and proliferation, and induces apoptosis by modulating several signaling pathways. However, further investigation is necessary to elucidate the mechanisms by which MPM-1 influences mitochondrial activity and, consequently, regulates various signaling pathways to impede tumor progression.

Rotenone, a compound recognized as a mitochondrial complex I inhibitor, is a toxic substance derived from leguminous plants and utilized as a botanical insecticide (Ochi et al., 2016). Research findings indicate that rotenone significantly suppresses the proliferation, invasion, and migration of colon cancer cells while promoting apoptosis via the PI3K/AKT signaling pathway (Xue et al., 2020). Additionally, it has been reported that rotenone plays a critical role in the modulation of Parkinson’s disease by inducing neuronal cell death and oxidative stress (Zhou et al., 2018; Johnson and Bobrovskaya, 2015). Recent investigations have also demonstrated that rotenone can impede the progression of various cancers, including lung and liver cancer, by disrupting autophagic flux and inhibiting cancer cell proliferation (Hu et al., 2016). Furthermore, rotenone has been shown to alter the radiation response and inhibit tumor growth in sarcomas and fibrosarcomas in murine models (Murley et al., 2017). It also inhibits the activation of STAT3 in lung cancer cells, thereby reducing cell survival and decreasing resistance to chemotherapy (Su et al., 2012). Moreover, rotenone induces reactive oxygen species (ROS)-mediated apoptosis in MCF-7 human breast cancer cells through the JNK and p38 signaling pathways (Deng et al., 2010). Consequently, rotenone has demonstrated anticancer properties by inducing apoptosis in various cancer cell types and inhibiting their proliferation and migration, suggesting its potential as a broad-spectrum anticancer agent. In the present study, rotenone was employed as a positive control to evaluate the anticancer efficacy of MPM-1. The results indicated that MPM-1 effectively inhibited tumor cell proliferation and migration while promoting apoptosis both *in vitro* and *in vivo*, thereby exerting its broad-spectrum anticancer effects through multiple pathways, comparable to those of rotenone.

In summary, we have synthesized mitochondrial fluorescent probes, specifically MPM-1, and validated its anticancer efficacy both *in vivo* and *in vitro*. Furthermore, we explored the molecular mechanisms underlying its tumor-suppressive properties through RNA sequencing. Notably, we innovatively demonstrated that MPM-1 exerts its broad-spectrum anticancer effects via multiple signaling pathways, including the MAPK, PI3K/Akt, Hippo, Wnt, and mTOR pathways. Our findings contribute to the development of novel strategies for cancer treatment and offer new insights into the discovery of antitumor agents that target mitochondrial functions.

## 4 Materials and methods

### 4.1 Cell culture

The non-small cell lung cancer cell lines A549 and H1299, as well as the colon adenocarcinoma cell lines SW480 and LOVO, were obtained from the American Type Culture Collection (ATCC). A549 and H1299 cells were cultured in RPMI 1640 medium, while SW480 and LOVO cells were maintained in Dulbecco’s Modified Eagle Medium (DMEM). All cultures were incubated at 37°C in a humidified atmosphere containing 5% CO2. Both RPMI 1640 medium (Catalog No. 23F301, Pricella) and DMEM (Catalog No. 24 × 681, Pricella) were supplemented with 1% penicillin and streptomycin (Catalog No. UB89609, BIOODIN) and 10% fetal bovine serum (Catalog No. UB68506T050, BIOODIN). To ensure the absence of *mycoplasma* contamination, all cell lines were subjected to testing using a *Mycoplasma* PCR kit, confirming their mycoplasma-free status.

### 4.2 Dilution and dosage of MPM-1

The MPM-1 powder was reconstituted in DMSO and preserved at the initial concentrations of 4 mM and 8 mM for future use. For the treatment of cells cultured in six-well plates, 2 µL of the various drug concentrations were extracted and subsequently added to 2 mL of the culture medium. In parallel, 2 µL of DMSO was incorporated into the corresponding control wells, with similar procedures applied for the volumes of the drug or control in the other well plates.

### 4.3 Cell activity measurement

Various concentrations of MPM-1 were applied to SW480, LOVO, A549, and H1299 cell lines utilizing 96-well plates, with a treatment duration of 24 h. The assessment of cell viability was conducted employing the Cell Counting Kit-8 (catalogue no. CK04, Dojindo), in accordance with the manufacturer’s instructions. Absorbance readings were obtained at a wavelength of 450 nm using an enzyme labeler (BioTek, USA).

### 4.4 Mito tracker green FM staining

Cells treated with MPM-1 for a duration of 24 h were cultured in NEST confocal cell culture plates. Following the fluorescent labeling of mitochondria using Mito Tracker Green FM (Catalog No. HY-135056, MedChemExpress), the nuclei were subsequently stained with Hoechst 33,342 (Catalog No. C0071S, Beyotime). Imaging was conducted utilizing a confocal microscope.

### 4.5 Cell cycle assay

The influence of MPM-1 on the cell cycle was assessed utilizing flow cytometry. Following fixation in cold 70% ethanol, cells were stained with propidium iodide (PI). The cell cycle analysis was conducted using the Cell Cycle Assay Kit (Catalog No. KGA512, KeyGEN Biotech, Nanjing, China), in accordance with the manufacturer’s instructions. Flow cytometry was employed to identify the labeled cells (Beckman, United States).

### 4.6 Colony formation

In 12-well plates, SW480, LOVO, A549, and H1299 cell lines were seeded at a density of 1,000 cells per well. Upon the formation of colonies consisting of four to 8 cells, different concentrations of MPM-1 were administered. The cell culture media was refreshed every 3 days. Following a 14-day incubation period, the cells were fixed using 4% paraformaldehyde and subsequently stained with crystal violet.

### 4.7 EdU assay

SW480, LOVO, A549, and H1299 cell lines subjected to treatment with MPM-1 24 h were subsequently stained utilizing the BeyoClick™ EdU-488 Kit (Catalog No. C0071S, Beyotime, Shanghai, China), in accordance with the protocols provided by the manufacturer.

### 4.8 Western blot

The SW480, LOVO, A549, and H1299 cell lines were administered MPM-1 for a duration of 24 h, after which protein extraction was performed followed by Western blot analysis. The Western blot analysis was conducted in accordance with the established protocols previously outlined (Shen et al., 2021). A comprehensive list of all primary antibodies utilized in this assay, which are specific to the corresponding antigens, can be found in Supplementary Table 1.

### 4.9 Wound healing assay

Following the inoculation of SW480, LOVO, A549, and H1299 cell lines in 6-well plates, a scratch assay was performed using a 10 µL needle tip. The cells were subsequently treated with MPM-1, and the resulting scratches were documented via microscopy (Eclipse Ti-S, Japan) after a 24 h incubation period. Cell motility was subsequently quantified utilizing ImageJ software.

### 4.10 Trans-well migration assay

The cell lines SW480, LOVO, A549, and H1299 were introduced into the upper chamber of Transwell chambers and cultured for a duration of 12 h in the presence of MPM-1. Following this incubation period, the cells were fixed using 4% paraformaldehyde, subsequently stained with crystal violet, and then examined microscopically for photographic documentation and cell counting.

### 4.11 Apoptosis assay

Following the inoculation of SW480, LOVO, A549, and H1299 cell lines in 6-well plates, the cells were subjected to treatment with MPM-1 for a duration of 24 h. Subsequently, the cell culture supernatant and the cells were harvested, washed through centrifugation with phosphate-buffered saline (PBS), and apoptosis was assessed utilizing the Annexin V-FITC Staining Kit (Catalog No. A211-02, Vazyme). The stained cells were then analyzed via flow cytometry (Beckman, United States).

### 4.12 RNA sequence

DMSO and MPM-1 were administered to SW480 cells for a duration of 24 h. Following the digestion and washing of the cells in phosphate-buffered saline (PBS), they were resuspended in Trizol reagent (ThermoFisher Scientific, United States). The library construction, RNA sequencing, and subsequent analysis were conducted by Beijing Biomarker Technology (Biomarker Beijing, China).

### 4.13 *In vivo* xenograft model

To establish a xenograft tumor model, six nude mice, aged 6–8 weeks, were randomly divided into three groups, with each mouse receiving a subcutaneous injection of 8 × 10^6^ cells. Beginning on the 14th day, the mice in each group were administered intraperitoneal injections every 3 days of either DMSO, MPM-1 (1 mg/kg), or Retenone (1 mg/kg), and tumor size was measured. Tumor volume (in mm^3^) was calculated using the formula: volume (mm^3^) = (width^2^ (mm^2^) × length (mm))/2. The mice were euthanized humanely via intraperitoneal injection of Avertin (Sigma-Aldrich, Shanghai, China), which was dissolved in tert-amyl alcohol and 0.9% saline, at a dosage of 0.2–0.4 mL per 10 g of body weight. For subsequent analysis, tumor, heart, liver, spleen, and lung tissues excised from the nude mice were fixed in formalin, embedded in paraffin, and sectioned.

### 4.14 Hematoxylin and eosin (HE) staining

Paraffin-embedded tissue sections were obtained from the heart, liver, spleen, lung, and kidney of murine subjects. These sections underwent a series of procedures, including dewaxing, hematoxylin-eosin staining, and dehydration.

### 4.15 Immunohistochemistry (IHC)

Tissue sections embedded in paraffin were obtained from tumors developed *in vivo*. Immunohistochemistry (IHC) was conducted in accordance with established protocols as detailed in prior research (Shen et al., 2021). The specific antibodies utilized in this study are enumerated in Supplementary Table 1.

### 4.16 Statistical analysis

Data analysis was conducted utilizing GraphPad Prism five software (GraphPad Software, San Diego, CA, United States). The results are presented as mean ± Standard Error of Mean (SEM). Statistical significance was assessed using t-tests for comparisons between two groups and one-way ANOVA for comparisons involving more than two groups, unless otherwise specified. P-values less than 0.05 were deemed statistically significant, with the following designations: *p < 0.05, **p < 0.01, ***p < 0.001, and ns indicating no statistically significant difference.

## Data Availability

The data presented in the study are deposited in the GEO repository, accession number GSE293903.
