# Supplementary material for: Synthesis of a novel mitochondrial fluorescent probe - killing cancer cells in vitro and in vivo
Source: Front Pharmacol. 2025 Apr 16;16:1543559. doi: 10.3389/fphar.2025.1543559 (PMC12040830; doi:10.3389/fphar.2025.1543559)
Supplement: Supplementary file 1 [file DataSheet1.docx]

Supplementary Material

Synthesis of a Novel Mitochondrial Fluorescent Probe - Killing Cancer Cells *in Vitro* and *in Vivo*

**Xiaowen Yang^1, #^，Yiting Zhan^1, #^, Yifei Li^1, 2^, Xinzhuang Shen^1, 2^, Yuqiu Ma^3^, Zongjun Liu^1, 2^, Yipeng Liu^1^, Chengjin Liang^1^, Xiaoyuan Zhang^1, *^, Yehao Yan^4, *^, Wenzhi Shen^1, *^**

^#^ Xiaowen Yang and Yiting Zhan contributed equally to this work.

*** Correspondence:** Wenzhi Shen: [shenwenzhi2011@126.com](mailto:shenwenzhi2011@126.com); Yehao Yan: [yanyehao_322@163.com](mailto:yanyehao_322@163.com); Xiaoyuan Zhang, [xiaoyuan0321smile@163.com](mailto:xiaoyuan0321smile@163.com).

**
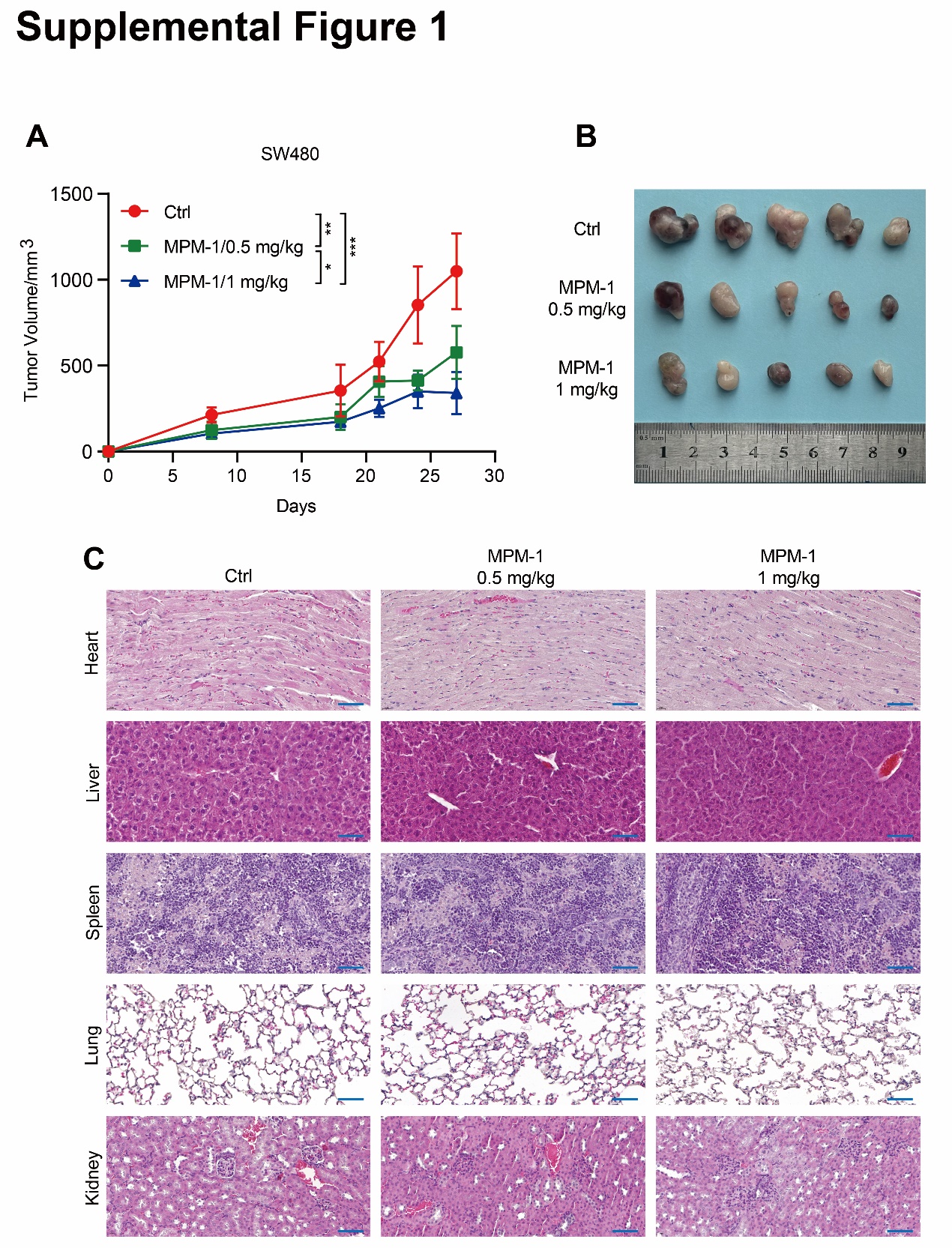
**

**Supplemental Figure 1.** MPM-1 inhibits colorectal cancer xenograft tumor progression *in vivo*

(**A**) SW480 cells were administered via injection into the fourth fat pad of nude mice. On the 15th day post-injection, three groups of mice received intraperitoneal injections of DMSO, MPM-1 (0.5 mg/kg), and MPM-1 (1 mg/kg), respectively, and tumor growth curves were subsequently generated. The sample size was N=5, and statistical significance was denoted as *p<0.05, **p<0.01, and ***p<0.001. (**B**) Tumors excised from the various groups of mice are presented. (**C**) Photomicrographs of HE staining of tissue sections from the heart, liver, spleen, lung, and kidney of the mice are displayed, with a scale bar of 50 μm.

**Supplemental Table 1**

| **Antibody** | | **Clone, Cat #** | **Vendor** | **City, State, Country** |
| --- | --- | --- | --- | --- |
| Ki67 | Rabbit monoclonal | Ab16667 | Abcam | Hong Kong, China |
| Caspase 3 | Rabbit monoclonal | 14220 | Cell Signal Technology | Danvers, MA, USA |
| Cleaved Caspase 3 | Rabbit monoclonal | 9661 | Cell Signal Technology | Danvers, MA, USA |
| Bax | Rabbit monoclonal | 60267-1-Ig | Proteintech | Wuhan, China |
| Bcl-2 | Rabbit monoclonal | 60178-1-Ig | Proteintech | Wuhan, China |
| Bcl-xl | Rabbit polyclonal | 26967-1-AP | Proteintech | Wuhan, China |
| β-actin | Mouse monoclonal | sc-47778 | Santa Cruz Biotechnology | Santa Cruz, CA, USA |
| Epithelial-Mesenchymal Transition (EMT) Antibody Sampler Kit | Rabbit monoclonal | 9782 | Cell Signal Technology | Danvers, MA, USA |
| Cyclin D1 | Rabbit monoclonal | 2978T | Cell Signal Technology | Danvers, MA, USA |
| Cyclin D3 | Mouse monoclonal | 2936T | Cell Signal Technology | Danvers, MA, USA |
| CDK6 | Mouse monoclonal | 3136T | Cell Signal Technology | Danvers, MA, USA |
| CDK4 | Rabbit monoclonal | 12790T | Cell Signal Technology | Danvers, MA, USA |
| Bax | Rabbit polyclonal | WL01637 | Wanleibio | Shenyang, China |
| P-ERK | Rabbit polyclonal | WLP1512 | Wanleibio | Shenyang, China |
| ERK | Rabbit polyclonal | WL01770 | Wanleibio | Shenyang, China |
| P-P38 | Rabbit polyclonal | 28796-1-AP | Proteintech | Wuhan, China |
| P38 | Mouse | 66234-1-Ig | Proteintech | Wuhan, China |
| P-JNK | Rabbit | 80024-1-RR | Proteintech | Wuhan, China |
| JNK | Mouse | 66210-1-Ig | Proteintech | Wuhan, China |
| P-YAP1 | Rabbit polyclonal | 29018-1-AP | Proteintech | Wuhan, China |
| YAP1 | Rabbit polyclonal | WL03624 | Wanleibio | Shenyang, China |
| P-PI3K | Rabbit | 4228S | Cell Signal Technology | Danvers, MA, USA |
| PI3K | Rabbit | 4292S | Cell Signal Technology | Danvers, MA, USA |
| P-AKT | Rabbit polyclonal | WLP001a | Wanleibio | Shenyang, China |
| AKT | Rabbit polyclonal | WL0003b | Wanleibio | Shenyang, China |
| P-mTOR | Mouse monoclonal | 67778-1-Ig | Proteintech | Wuhan, China |
| mTOR | Mouse monoclonal | 66888-1-Ig | Proteintech | Wuhan, China |
